# Allergenic potential of ornamental Cupressales species and its consequences for urban planting

**DOI:** 10.1038/s41598-026-40332-w

**Published:** 2026-02-17

**Authors:** Oliwia Wieczorek, Agata Frątczak, Łukasz Grewling

**Affiliations:** 1 Department of Systematic and Environmental Botany, Faculty of Biology, Adam Mickiewicz University, Uniwersytetu Poznańskiego 6, 61-614 Poznań, Poland; 2 Laboratory of Aerobiology, Faculty of Biology, Adam Mickiewicz University, Uniwersytetu Poznańskiego 6, 61-614 Poznań, Poland

**Keywords:** Cup a 1, ELISA, Pollen allergy, Environmental monitoring, Biological air pollutants, Phenology, Ecology, Ecology, Environmental sciences, Plant sciences

## Abstract

Cupressales species are widely planted in urban landscapes, where their pollen represents a growing source of biological air pollution and contributes to seasonal allergies. Linking their flowering dynamics with atmospheric allergen loads is essential for understanding ecological flows of airborne particles in cities. We aimed to quantify interspecific differences in allergenic potential among common urban Cupressales taxa and to determine how flowering periods and pollen production shape airborne allergen levels. We specifically assessed variation in Cup a 1 homolog abundance to evaluate its relevance for landscape-scale allergy risk. The study was conducted in Poznań (Poland) during the 2023–2024 pollen seasons. Phenological observations, aerobiological monitoring, and immunoenzymatic assays were combined to relate flowering timing, atmospheric pollen concentrations, and allergen content across ten Cupressales species. Species differed substantially in flowering phenology, pollen output, and allergen levels. *Taxus baccata, Thuja occidentalis*, and *Th. plicata* released large amounts of pollen but contained low levels of Cup a 1 homologs. Conversely, *Juniperus* taxa and *Callitropsis nootkatensis* showed consistently high allergenicity, contributing disproportionately to peak atmospheric allergen loads despite moderate pollen release. These patterns indicate that allergen exposure risk is driven not only by pollen abundance but also by species-specific allergen potency. Urban planting dominated by high-allergenicity taxa may therefore intensify allergy symptoms at the neighbourhood scale. Our findings demonstrate that allergenicity metrics can inform urban vegetation planning. Selecting species with favourable phenological and allergenic profiles may help reduce airborne allergens and support healthier, more resilient urban landscapes.

**Supplementary Information:**

The online version contains supplementary material available at 10.1038/s41598-026-40332-w.

## Introduction

Cupressales is an order of coniferous plants (including two major families, Cupressaceae and Taxaceae) that are commonly found in temperate regions. In Europe, representatives of this group include genera such as *Cupressus* (cypress), *Juniperus* (juniper), *Thuja* (arborvitae), *Chamaecyparis* (false cypress) and *Taxus* (yew)^1^. Taxa belonging to Cupressales are widely used as ornamental plants in horticulture and urban landscaping, owing to their advantageous traits, including year-round greenery, tolerate pruning and shaping, good resistance to air pollution and drought^2,3^. Numerous new cultivars have been developed, further increasing their popularity. However, their widespread use has an important downside—pollen allergy.

Pollen grains released by species of the order Cupressales are a significant cause of seasonal allergies worldwide^4–6^. The prevalence of Cupressales pollen allergy in the general population usually ranges from 2 to 4% in Southern European countries to as much as 13% in Japan. Among individuals with allergic diseases, however, the proportion of sensitized people is 14–32%, showing a clear upward trend in recent decades^7,8^. In Mediterranean countries, the emission of Cupressales pollen, primarily from the Arizona cypress (*Cupressus arizonica*) and Italian cypress (*Cupressus sempervirens* L.), is a major source of winter allergies, when other plants are not in bloom^9,10^. The allergenic significance of Cupressales pollen has been confirmed in studies conducted in France^11–13^, Spain^14,15^, Portugal^16^, Italy^17,18^, Greece^19^, Israel^20^ and Turkey^21^. Their pollen grains also pose a serious problem in Japan, where pollen from Japanese cedar (*Cryptomeria japonica*) is the primary airborne allergen, affecting up to 25% of the population seasonally^22^. Another significant source of allergenic pollen in Japan is the Hinoki cypress (*Chamaecyparis obtusa*)^23^. Meanwhile, in the southern region of North America, three *Juniperus* species, i.e. cade juniper (*Juniperus oxycedrus*), mountain cedar (*Juniperus ashei*), and eastern red cedar (*Juniperus virginiana*) are significant contributors to seasonal allergies^24,25^.

The primary allergens (group 1) in pollen grains of species within this order are proteins with a molecular weight of 40–47 kDa, exhibiting pectin lyase activity^26,27^. This enzyme is believed to be responsible for pectin degradation during pollen tube growth^28^. In the WHO/IUIS Allergen Nomenclature Database eight species from Cupressales are listed containing pectate lyase type allergen, i.e. *Ch. obtusa* (Cha o 1), *C. japonica* (Cry j 1), *C. arizonica* (Cup a 1), *C. sempervirens* (Cup s 1), *J. ashei* (Jun a 1), *J. oxycedrus* (Jun o 1) and *J. virginiana* (Jun v 1). The presence of major allergens in pollen grains clearly indicates their potential to cause allergic reactions. However, the allergenicity of pollen is also strongly influenced by the quantitative content of allergens within the grains. Studies analyzing the variability of major pollen allergens in other taxa, such as grasses and birches, have demonstrated significant differences in allergen content even among closely related species^29,30^. To date, such a comparison has not been conducted within any species of the Cupressales order.

The analysis of airborne pollen concentrations of different Cupressales species is challenging due to their close morphological similarity, which prevents genus-level identification^31,32^. In general, Cupressales pollen grains are small (20–30 μm), round, and characterized by a thin exine and a very thick intine, often giving the interior of the grain a distinctive star-shaped appearance. Additionally, the exine surface is covered with small, irregularly scattered granules known as orbicules (Ubisch bodies). Since these features are common to most Cupressales pollen grains, standard aerobiological analyses (quantifying airborne pollen concentrations) typically group the pollen of Cupressaceae and Taxaceae together as ‘Cupressales’^33^. This grouping has further implications, as detailed pollen calendars illustrating the pollination periods of individual Cupressales species are scarce^34^. It is important to note that the Cupressales pollen season is long, lasting 2–3 months, and consists of several distinct peaks^7,34^ each associated with the pollination of specific species. However, which species contribute most significantly to these peaks and the allergenic potential of pollen during each peak remain unknown. This lack of detailed knowledge makes it difficult to accurately assess the risk of pollen allergies at specific times within the Cupressales pollen season.

Therefore, our study aimed to address this gap by examining the variability in the quantity of homologs of major pollen allergens in the most common ornamental Cupressales species in Central Europe, particularly in Poznań, Poland. By integrating this data with phenological information (pollination period) and airborne pollen concentration measurements, we identified taxa with the highest allergenic potential, posing the greatest risk to sensitized allergic individuals.

## Materials and methods

### Plant material and study area

The study focused on 10 species of Cupressales: English yew (*Taxus baccata*), Nootka cypress (*Callitropsis nootkatensis*), Lawson’s cypress (*Chamaecyparis lawsoniana*), northern white cedar (*Thuja occidentalis*), western red cedar (*Thuja plicata*), savin juniper (*Juniperus sabina*), eastern red cedar (*Juniperus virginiana*), hybrid juniper (*Juniperus x pfitzeriana*), common juniper (*Juniperus communis*), and Chinese juniper (*Juniperus chinensis*).

Aerobiological monitoring, phenological observations, and pollen grain collection were conducted during two Cupressales pollen seasons (2023–2024) in Poznań, Poland (52° 24′ N, 16° 56′ E). The city is located in a temperate climate zone. The highest mean monthly temperatures are recorded in July (18.1 °C), while the lowest occur in January (− 1.6 °C). Annual precipitation ranges from 500 to 550 mm^35^. Meteorological data were collected from stations belonging to the Polish Institute of Meteorology and Water Management, located at Ławica Airport.

### Phenological observations

**Fig. 1:**
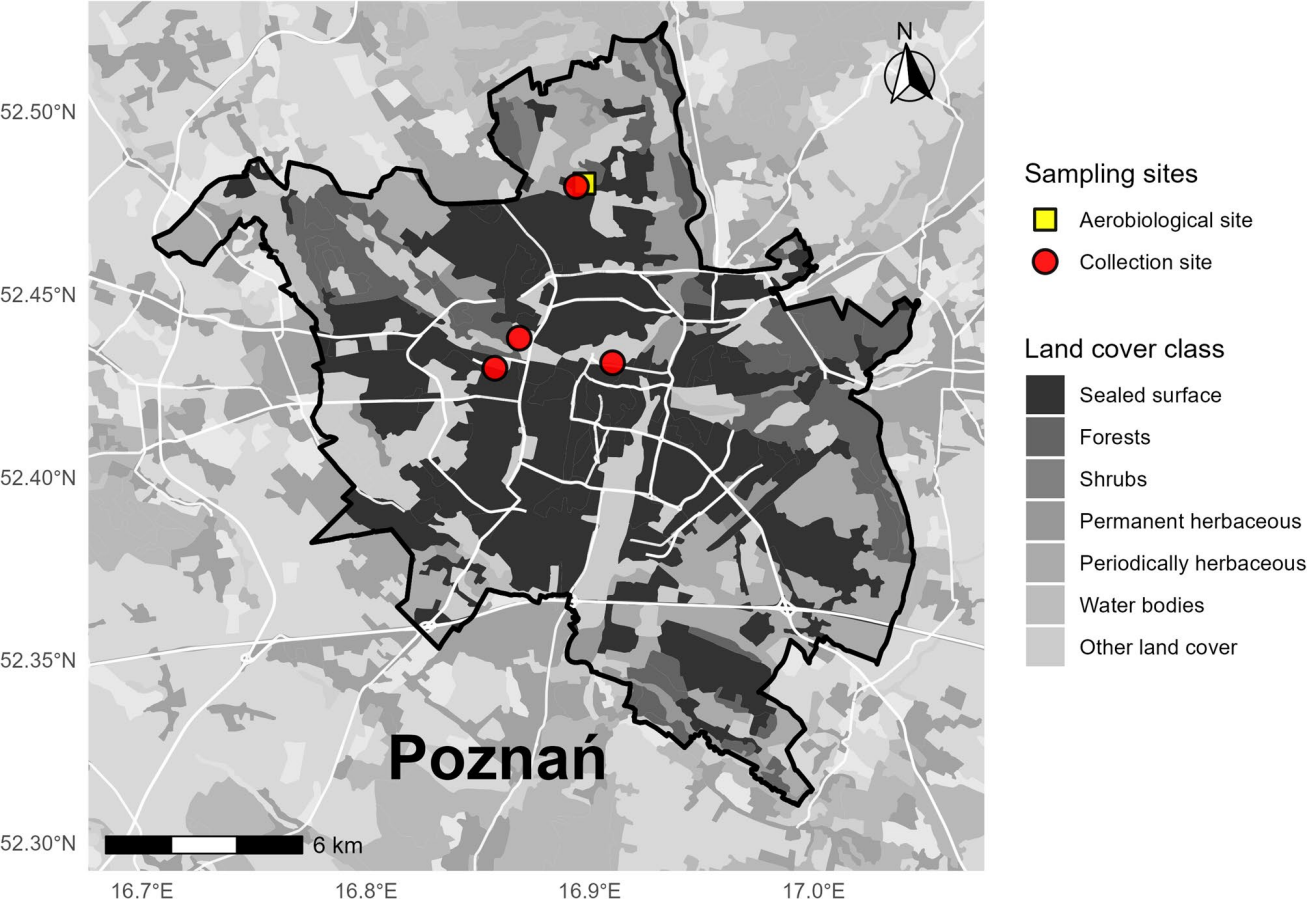
Location of the aerobiological station (yellow square) and pollen collection/phenological observation sites (red circles) within the city of Poznań (Poland). The map presents the administrative boundary of the city and general land cover classes for spatial context.

Observations of pollination process were conducted once a week from early February to late May. In 2023, six Cupressales species were monitored: *T. baccata, Th. occidentalis, Th. plicata, Ch. lawsoniana, J. communis,* and *J. sabina.* In 2024, in addition to these species, phenological observations were extended to four additional species: *C. nootkatensis, J. virginiana, J.* × *pfitzeriana,* and *J. chinensis.* The observations were carried out in the Botanical Garden of Adam Mickiewicz University, Dendrological Garden of the University of Life Sciences, Campus Morasko, and Cytadela Park in Poznań (Fig. 1). Detailed information on the number of species studied at each site is provided in Supplementary Table S1. Three to five individuals per species were observed. In practice, for each individual during each field visit, approximately 20–30 microsporophylls were assessed every 2–3 days, randomly selected from the available male shoots. Based on the percentage of shedding microsporophylls, three major pollen release stages were distinguished: (1) beginning (Stage 1), when 10% of microsporophylls released pollen, (2) peak (Stage 2), when 50% of microsporophylls released pollen grains, and (3) end of pollination (Stage 3), when the pollen release ended and fruit set became visible^36^.

### Aerobiological monitoring

Pollen samples used for atmospheric pollen content analysis were collected using a Hirst-type volumetric trap (Burkard Manufacturing Co Ltd) located on the roof of the Faculty of Biology at Adam Mickiewicz University (7.5 km from the city center) at an altitude of 18 m (a.g.l.). The volumetric sampler operates by continuously drawing in 10 L of air per minute. The internal drum rotates at a speed of 2 mm/hour, allowing for hourly pollen grain analysis. A Melinex tape coated with an adhesive is affixed to the drum’s surface, where airborne particles, including pollen grains, are deposited. The device operates on a 7-day cycle. At the end of each week, the tape was cut into seven equal Sects. (14 mm × 48 mm), each representing one day, and stained with fuchsin solution. The preparations were then examined under a light microscope at 400 × magnification.

Daily pollen concentration was calculated using the four-horizontal-transects method according to aerobiological standards^37^. The obtained value was multiplied by a factor accounting for the total slide surface, the analyzed area on the slide, and the volume of analyzed air^38^. The result was expressed as pollen grains per cubic meter per day (pollen grains/m^3^/day). To determine the duration of the Cupressales pollen season, the 95% cumulative percentage method was applied. According to this method, the season starts on the day when the daily pollen concentration reaches 2.5% of the total annual pollen sum and ends when it reaches 97.5%^39^. The obtained pollen concentration data were then compared with the pollination periods of individual species.

### Immunoenzymatic methods

Pollen was collected from flowering individuals that were also included in phenological observations, from the same locations, using a laboratory sieve. Due to the generally low pollen yield and the relatively large amount of pollen required for ELISA analyses, samples from individual species were pooled within the same season. After collection, pollen grains were stored in a refrigerator at –20 °C. To obtain the allergen solution, 0.1 g of pollen was extracted in 1.5 ml of extraction buffer (NH₄HCO₃, H₂O, NH₃). To quantify the Cup a 1 allergen homologs released from pollen grains, a sandwich-type enzyme-linked immunosorbent assay (ELISA) was performed using anti-Cup a 1 monoclonal antibodies and biotinylated monoclonal antibodies supplied by ROXALL GROUP. Streptavidin-peroxidase (Sigma-Aldrich S5512) was used as the enzyme, while 3,3′5,5′-tetramethylbenzidine (Sigma-Aldrich T0440) served as the substrate. The reaction was stopped by adding 2.5 M H₂SO₄ (5 N). The concentration of Cup a 1 in the sample was measured by detecting absorbance at 450 nm. Using a hemocytometer, the number of pollen grains used for sample preparation was counted, and the concentration of Cup a 1 allergen homologs per pollen grain was calculated, allowing for the determination of allergenicity (pg Cup a 1/pollen grain) of selected Cupressales species. Because the samples were pooled within each season, only interannual differences in pollen allergenicity could be assessed (2023 vs. 2024), with no within-season replication. Therefore, no formal statistical analysis of interspecific variability was performed.

## Results

### Weather background

In 2023, the average temperature in Poznań was 10.6 °C, while in 2024, it increased to 12.3 °C. Annual precipitation and average wind speed were similar in both years—526 mm and 3.7 m/s in 2023, and 534 mm and 3.6 m/s in 2024. Between February and May, corresponding to the pollination period of the observed Cupressales species, total precipitation was 31.5 mm in 2023, whereas in 2024 it increased to 169.2 mm. The average daily temperature during this period was 7.1 °C in 2023 and 10.2 °C in 2024.

### Phenological observation of pollination

Among the species studied*, T. baccata* began pollinating the earliest in both years (Supplementary Table S2). The first open inflorescences (Stage 1) of *T. baccata* in Poznań were observed on February 16, 2023, and February 10, 2024. The peak pollen release (Stage 2) occurred during the first three weeks of March in 2023 and at the turn of February and March in 2024. The pollination period of *T. baccata* ended (Stage 3) on March 27, 2023, and March 9, 2024. The pollination periods of the two observed *Thuja* species overlapped and were mainly limited to March in 2023, and from mid-February to early March in the following year. The Stage 2 phase was recorded in mid-March during the first year of observation and in early March in 2024. On April 1, 2023, pollen release of *Juniperus sabina* began, with the season ending on April 20, 2023. The Stage 2 phase was recorded between the second and third weeks of April. Similar to the other species studied, the pollination period in 2024 started much earlier than in 2023, with the Stage 2 phase occurring in the second and third weeks of March. *J. communis* was the latest species to pollinate, with the peak of pollen release in the first week of May 2025 and the beginning of April 2025. In 2024, three other *Juniperus* species were observed: *J. chinensis, J. virginiana*, and J. × *pfitzeriana.* All of them pollinated just before *J. sabina*, predominantly in the first part of March. In 2024, the pollination period of *Callitropsis nootkatensis* started on February 25 and ended on March 15, with the peak occurring between the first and second weeks of March. *Chamaecyparis lawsoniana* is considered a late-flowering species, releasing pollen in the second half of April in 2023 and the second half of March in 2024. Overall, the sequence of pollination among the observed species was similar in both years. It began with *Taxus baccata*, followed by the two *Thuja* species, while *Juniperus* and *Chamaecyparis* pollinated later in the season. *Juniperus communis* was the last species to pollinate.

### Airborne pollen concentration and its relevance to phenological data

In 2023, the first Cupressales pollen grains were detected in the air in mid-January. The pollen season began on March 4, ended on May 11, and lasted 69 days. The total sum of pollen grains in the air was 2,280 (pollen grains/ m^3^). The highest daily pollen concentration (308 pollen grains/m^3^/day) was observed on March 23 (Table 1). The pollen season also had several smaller peaks, specifically on March 14 (298 pollen grains/m^3^/day), March 19 (210 pollen grains/m^3^/day), and April 10 (227 pollen grains/m^3^/day).

**Table 1:** Main characteristics of Cupressales pollen seasons (2023–2024).

Characteristics	Season 2023	Season 2024
Start of pollen season	Mar-04	Feb-17
End of pollen season	May-11	Apr-15
Pollen season duration (days)	69	61
Total sum of pollen (pollen/m^3^)	2280	2345
Maximum daily pollen level (pollen/m^3^)	308	262
Day with maximum pollen level	Mar-23	Mar-02

In 2024, the first pollen grains in the air were also observed in mid-January. The pollen season began on February 17, ended on April 15, and lasted 61 days. The total sum of pollen grains in the air was 2,345 (pollen grains/ m^3^). The highest daily pollen concentration (262 pollen grains/m^3^/day) was recorded on March 2 (Table 1). The 2024 Cupressales pollen season also had several smaller peaks, occurring on February 23 (164 pollen grains/m^3^/day), March 10 (119 pollen grains/m^3^/day), and March 15 (198 pollen grains/m^3^/day).

**Fig. 2:**
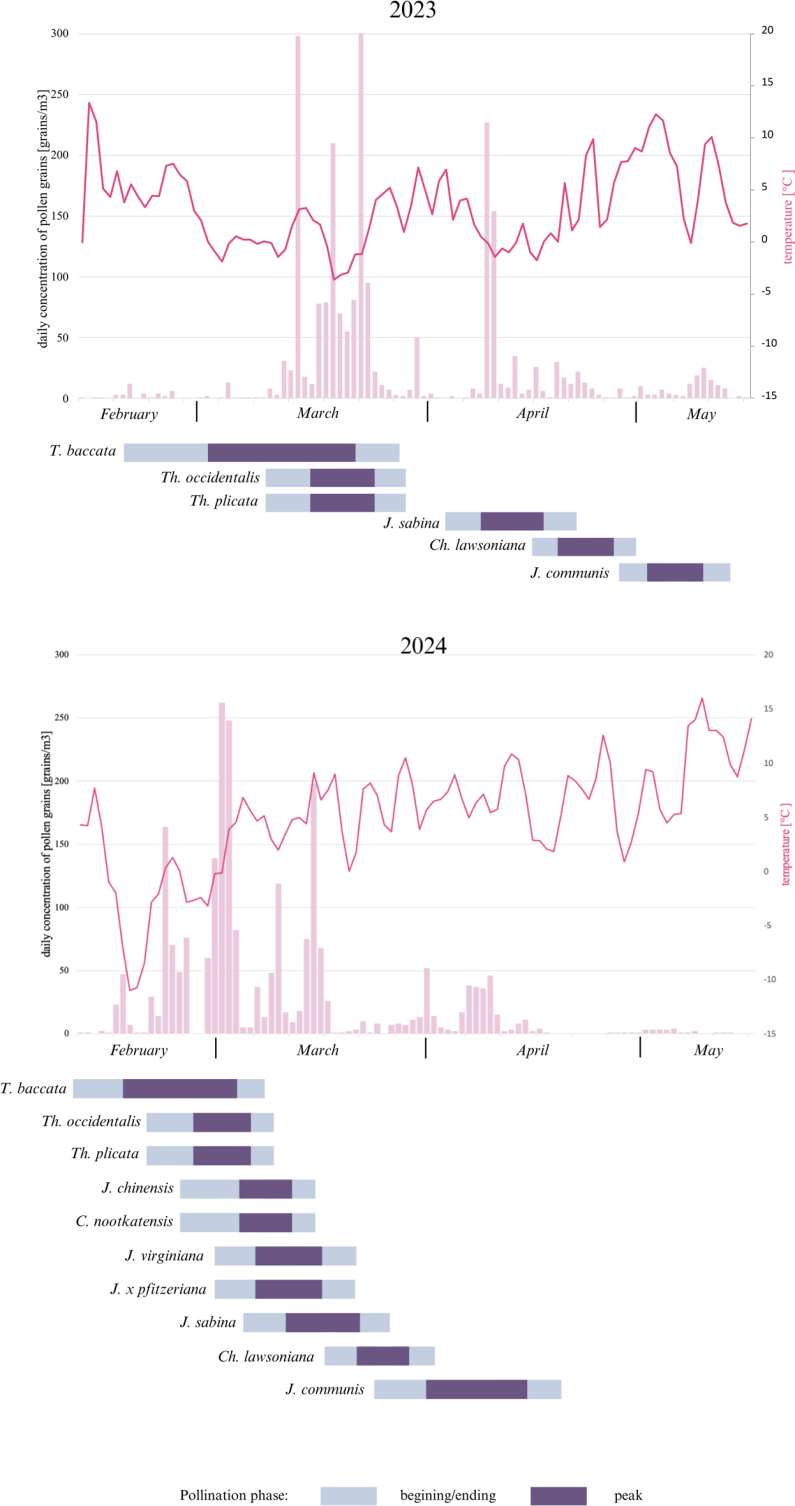
Concentration of Cupressales pollen (pink color) compared to the pollen calendar of selected Cupressales species in Poznań, Poland, 2023–2024.

Based on flowering observations and airborne pollen grain data (Fig. 2), the species contributing most significantly to the seasonal pollen sum were *T. baccata, Th. occidentalis,* and *Th. plicata.* In the first half of April 2023, a distinct peak in pollen grain concentration corresponded with *J. sabina* pollen release. In 2024, this peak also matched the pollen release periods of other junipers, namely *J. virginiana* and *J. x pfitzeriana.* Other species, such as *J. communis*, had a smaller impact on the total pollen sum in the air but significantly prolonged the Cupressales pollen season due to their late flowering period.

### Allergenic protein homolog content

The pollen grains of the studied species exhibited significant variations in the amount of allergenic Cup a 1 homolog (Table 2). The highest allergenicity was observed in the *C. nootkatensis*, with a mean of 2.085 pg/pollen grain. Relatively high values were also recorded for the *Juniperus* genus, specifically *J. virginiana* (1.006 pg/pollen grain), *J. communis* (0.964 pg/pollen grain), *J. x pfitzeriana* (0.879 pg/pollen grain), *J. sabina* (0.836 pg/pollen grain), and *J. chinensis* (0.471 pg/pollen grain). For the remaining species, namely *Th. plicata, Th. occidentalis, T. baccata,* and *Ch. lawsoniana*, Cup a 1 homologues were detected at values near or below the detection limit of the ELISA system used.

**Table 2:** Cup a 1 homolog content in selected Cupressales species. “N/A” (Not Available) denotes that the measurement was not performed for that given sample. “BLQ” (Below Limit of Quantification) indicates that the results were below the quantification limit of the method. Dash (–) indicates that the mean could not be calculated due to mathematical reasons, as the results were below the detection limit.

Species	Cup a 1 homolog content (pg/pollen grain)		
	Season 2023	Season 2024	Mean
*Callitropsis nootkatensis*	2.432	1.738	2.085
*Chamaecyparis lawsoniana*	0.004	BLQ	0.004
*Juniperus virginiana*	N/A	1.006	1.006
*Juniperus communis*	0.956	0.971	0.964
*Juniperus x pfitzeriana*	0.663	1.094	0.879
*Juniperus sabina*	1.048	0.624	0.836
*Juniperus chinensis*	0.257	0.658	0.471
*Taxus baccata*	0.014	BLQ	0.014
*Thuja occidentalis*	BLQ	BLQ	–
*Thuja plicata*	0.082	BLQ	0.082

## Discussion

### Allergenic potential of cupressales pollen

Observations that pollen allergenicity can vary substantially even among closely related species highlight the need to integrate molecular and proteomic approaches into classical aerobiological studies. To date, such complementary analyses have been conducted for several major allergenic taxa, including Poaceae^30,40^, Betulaceae^29,41^, and Asteraceae^42,43^. However, despite the clinical relevance of Cupressales pollen, variability in its allergenicity remains poorly characterized. Previous studies typically included only a limited number of species (2–3) and relied on semi-quantitative methods (e.g., SDS-PAGE, Western blotting)^44,45^ or indirect approaches (e.g., inferring allergen levels from the relationship between airborne pollen counts and measured allergen concentrations)^15^. Although these studies produced encouraging results—suggesting true species-specific differences in allergenicity within Cupressales—comprehensive and robust investigations have been lacking.

Here, we provide a systematic quantification of homologs of the major cypress pollen allergen, Cup a 1, across multiple Cupressales representatives. Using an enzyme-linked immunosorbent assay (ELISA), we determined allergenic protein levels in pollen grains from several species within the order. In the present study, high levels of proteins cross-reacting with Cup a 1 were detected in all examined juniper species, including *Juniperus sabina, J. virginiana, J. communis, J*. x *media*, and *J. chinensis*. Similarly, very high amount of Cup a 1-homologue was noted in *Callitropsis nootkatensis*, which is closely related to *Juniperus* and *Cupressus* genera^46^. In contrast to the other species investigated, the group I allergen was detected at much lower levels in the pollen of yew (*Taxus baccata*), false cypress (*Chamaecyparis lawsoniana*) and two species of *Thuja*. Arilla et al.^27^ showed that the antibodies developed for ELISA were capable of recognizing pectin lyase not only from *Cupressus*, but also *Juniperus* and *Thuja.* In general, in the Cupressaceae family, allergenic proteins exhibit a high degree of sequence identity (≥ 75%) and similarity (≥ 95%)^47,48^. Pectin lyase activity has been most extensively studied in Japanese cedar (*Cryptomeria japonica*), where cross-reactivity was demonstrated between the Cry j 1 allergen and the major allergens of Arizona cypress (*Cupressus arizonica*)—Cup a 1 and Mediterranean cypress (*Cupressus sempervirens*)—Cup s 1^49,50^. A highly homologous protein has also been characterized in Ashe juniper (*Juniperus ashei*)—Jun a 1^26^, prickly juniper (*Juniperus oxycedrus*)—Jun o 1 (EC 4.2.2.2), and eastern red cedar (*Juniperus virginiana*)—Jun v 1^51^. Cross-reactivity was also demonstrated between the Cry j 1 allergen and the major allergens of Hinoki cypress (*Chamaecyparis obtusa*)—Cha o 1^49,50^. This suggests that the differences in the level of Cup a 1 homologs in investigated species is related to the immanent characteristic of these pollen grains rather than differences in allergen-antibody recognizing efficiency.

The situation for *Taxus baccata* is less clear, as no previous data were available on the structure or cross-reactivity of its allergens. *T. baccata* belongs to the Taxaceae family and is therefore phylogenetically more distant from the other investigated taxa, which belong to the Cupressaceae family. However, Cupressaceae group 1 allergens form a highly conserved protein family with strong sequence and structural similarity, and numerous studies have shown that these allergens are functional homologs with high IgE cross-reactivity, with differences being largely quantitative rather than qualitative^47^. Consequently, antibodies raised against Cup a 1 are generally expected to recognize shared epitopes across group 1 allergens from different Cupressaceae taxa. To address potential concerns regarding a more phylogenetically distant taxon such as *T. baccata,* we sequenced its group 1 allergen and compared it with Cup a 1 (Supplementary Fig. S1). This analysis revealed a high amino acid similarity (94%), comparable to or even higher than similarities reported between Cup a 1 and some Cupressaceae genera^48^. These results indicate that the Taxus group 1 allergen should be efficiently recognized by anti–Cup a 1 antibody.

From this perspective, it is also important to note that phylogenetic relatedness is not always a reliable predictor of allergen similarity or ELISA cross-reactivity. For example, as shown by Radauer et al.^52^, highly conserved allergenic proteins can be present in species that are relatively distant phylogenetically, while closely related species may show significant divergence in specific allergen sequences. Antibody binding depends primarily on the conservation and surface accessibility of specific linear or conformational epitopes, which may remain preserved even between phylogenetically distant taxa. Moreover, protein folds can remain highly conserved despite substantial sequence divergence^53,54^. Therefore, although *Taxus* (Taxaceae) is taxonomically distant from *Juniperus* and *Thuja* (Cupressaceae), structurally constrained Cupressales group 1 allergens (Cup a 1–type pectate lyases) may still share conserved epitopes across both families. This interpretation is consistent with our sequence results and provides a plausible explanation for the observed ELISA reactivity, where differences in ELISA signals likely reflect true quantitative differences in allergen content.

### Pollen concentration and pollination phenology

Thanks to the combination of direct pollination observations and aerobiological data, we were able to determine which Cupressales species contributed most significantly to atmospheric pollen levels. Based on the collected data, it is evident that the primary sources of pollen are *Taxus baccata*, *Thuja occidentalis*, and *Th. plicata*. These species flower earliest in the season, with their pollination period spanning from mid-February to the end of March. Fortunately for allergy sufferers, *T. baccata, Th. occidentalis*, and *Th. plicata* exhibited the lowest levels of Cup a 1 homologs. In contrast, high concentrations of airborne pollen were recorded during the flowering of *Juniperus sabina, J. virginiana,* and *J.* × *pfitzeriana*, which showed relatively high pollen allergenicity among all taxa studied. The widespread planting of these species—particularly *J. sabina* and *J*. × *media*—combined with their high allergenic potential, suggests that they may pose a significant health risk to residents of Poznań, especially in the second part of Cupressales pollen season (April–May).

Whether the observed variation in pollen contribution is due to differences in pollen production or species abundance and distribution requires further investigation. In Central and Western Poland, only two native Cupressales species occur: *Taxus baccata* and *Juniperus communis*^55^. Both are relatively scarce, particularly *T. baccata*, which persists mainly at a few natural sites and is considered endangered. In forests, yew occurs singly or in small groups in the understory, limiting its pollen emission and dispersal^56^. *J. communis* is more widespread, typically growing as an understory species on poor, sandy podzolic and brown soils dominated by pine and pine–spruce stands^57^. However, its greater abundance does not translate into higher airborne pollen concentrations. In Poznań, pollen levels during the second part of the Cupressales season (flowering time of *Juniperus*) are much lower than in February–March, when yew releases pollen. Therefore, the high Cupressales pollen concentrations observed in the city are likely driven mainly by ornamental and planted taxa in the urban environment (parks and private gardens), rather than by native populations.

In our study, botanical gardens served as reference sites for phenological observations of well-identified Cupressales taxa, complemented by monitoring at several additional urban locations. The onset and peak of pollen release across the city were broadly consistent with the botanical garden records. Although some spatial variation between sites can be expected due to microclimatic differences—for example, as observed for birch in Poznań^58^—this variation is unlikely to exceed 1–2 weeks. Given that the Cupressales pollen season in Poznań lasts almost four months (February–May) and is characterized by several distinct peaks, we assume that such microclimatic shifts in flowering timing are too small to significantly affect the overall seasonal pattern.

Studies on the genus *Cupressus* have shown that pollen production can vary substantially between species. For example, *C. macrocarpa* produces significantly more pollen—nine times more than *C. arizonica* and 18 times more than *C. sempervirens*^59^. Comparable interspecific variation in pollen production has also been reported in other genera, such as *Artemisia. A. campestris*, for instance, produces considerably fewer pollen grains per plant compared to *A. vulgaris* and *A. absinthium*. Nevertheless, due to its widespread presence in rural environments and relatively high allergenicity^43^, *A. campestris* contributes substantially to overall airborne pollen loads and poses an increased risk to sensitized individuals in certain areas^60^.

Without such fundamental data, it is not possible to accurately assess the allergenic potential of specific plant species. While simplified pollen calendars fulfill general public needs, they are often insufficient for urban-scale assessments, where pollen exposure is highly localized. Greater efforts are needed within the scientific community to rigorously evaluate the allergenic characteristics of key species. Remarkably, even after decades of research, essential data—such as pollen production rates and allergen content per pollen grain—remain unknown for many highly allergenic taxa.

### Species selection for urban planting

The results of this study suggest that yews (*Taxus* spp.) and thujas (*Thuja* spp.) could be favorable choices for urban greening projects in terms of reducing exposure to group 1 allergens. However, further research is needed before these taxa can be confidently recommended as low-allergenic species for urban plantings. It should be borne in mind that other allergenic proteins not investigated in this study may be present in their pollen. In addition, published studies indicate that yew and thuja pollen may indeed cause allergic reactions^61,62^. Nevertheless, in comparison, to other investigated species, yew and thujas exhibited very low levels of the investigated pectin lyase allergens. An additional advantage of yews is their dioecious nature^1^, allowing for the selective planting of female individuals, which do not produce pollen, thereby further reducing airborne pollen exposure in urban environments Similarly, when selecting junipers (*Juniperus* spp.), it would be beneficial to prefer dioecious species, wherever possible. Although not all analyzed juniper species are dioecious, preference should be given to those that are (*J. sabina* and *J. communis*), as this also enables the strategic planting of female plants to minimize pollen emissions.

Beyond species selection, other considerations are critical for allergen-sensitive urban greening. Monocultures and dense hedgerows composed of highly allergenic species should be avoided, as they can lead to locally elevated pollen concentrations^63,64^. A diverse plant palette should be promoted, incorporating species and cultivars known for low pollen production and minimal allergenic potential. Trees with broad leaves and lower pollen dispersal, such as *Acer* (mapple) or *Tilia* (linden) can serve as effective buffer species, especially when placed strategically to intercept airborne allergens from more potent sources^65^. Additionally, the introduction of highly allergenic exotic species, such as *Cryptomeria japonica*^22^, which recently has been increasingly available in commercial garden shops (own observations), should be avoided to prevent the introduction of new allergenic sources into urban areas.

Overall, the findings highlight the importance of incorporating allergenicity into urban planting guidelines. Strategic selection of plant species based on their reproductive biology, flowering phenology, and allergenic cross-reactivity, combined with diversification of urban greenery, can significantly contribute to the creation of healthier urban environments and the improvement of life quality for allergy-prone individuals.

## Conclusion

Many factors influence how healthy and resident-friendly an urban space is, and one of them—often overlooked—is the allergenic potential of plants. As our research demonstrates, even closely related plant species can differ significantly in their allergenicity, pollination periods, and contribution to the total airborne pollen load. These differences can directly impact the severity and frequency of allergic symptoms among the population.

Our findings highlight the need for continued research, particularly to assess additional factors such as pollen production, spatial distribution, and plant abundance within urban environments. Furthermore, given the wide range of cultivars among ornamental plants, their allergenic properties should be evaluated prior to commercial distribution. Introducing standardized labelling of a species’ allergenic potential could offer valuable guidance to both landscape planners and individual buyers, helping to mitigate unintended health risks associated with urban greening.

## Supplementary Information

Below is the link to the electronic supplementary material.

Supplementary Material 1.

## Data Availability

The datasets used and/or analysed during the current study are available from the corresponding author on reasonable request.
