# Supplementary material for: Allergenic potential of ornamental Cupressales species and its consequences for urban planting
Source: Sci Rep. 2026 Feb 17;16:8887. doi: 10.1038/s41598-026-40332-w (PMC12987948; doi:10.1038/s41598-026-40332-w)
Supplement: Supplementary file 1 — Supplementary Material 1. [file 41598_2026_40332_MOESM1_ESM.pdf]

Supplementary Materials

Allergenic potential of ornamental Cupressales species and its consequences for urban planting

Oliwia Wieczorek[1], Agata Frątczak[1*], Łukasz Grewling[1,2]

[1]Department of Systematic and Environmental Botany, Faculty of Biology, Adam Mickiewicz University, Poznań, Poland

[2]Laboratory of Aerobiology, Faculty of Biology, Adam Mickiewicz University, Poznań, Poland

* Correspondence to: Agata Frątczak, Department of Systematic and Environmental Biology, Faculty of Biology, Adam Mickiewicz University, Uniwersytetu Poznańskiego 6, 61-614 Poznań, Poland, email: agataf@amu.edu.pl, phone number: +48 618295693

Table S1. The list of plant species investigated at each study site.

Site	Species
Botanical Garden	*C. nootkatensis, Ch. lawsoniana, J. sabina, J. virginiana, J. chinensis, J. x pfitzeriana, T. baccata, Th. plicata, Th. occidentalis*
Dendrological Garden	*J. communis*
Campus Morasko	*C. nootkatensis, Ch. lawsoniana, J. communis, J. sabina*
Cytadela Park	*J. chinensis, T. baccata, Th. plicata, Th. occidentalis*

Table S2. Pollen release stages of selected Cupressales species in seasons 2023-2024. "N/A" (Not Available) denotes that the measurement was not performed for that given sample. The presented values are averages calculated across the studied locations; see Supplementary Table S1.

Species	Year	Start of Pollination (Stage 1)	Peak (Stage 2)	End of Pollination (Stage 3)
Taxus baccata	2023	February 16	March 1 – March 20	March 27
	2024	February 10	February 19 – March 2	March 9
Thuja occidentalis	2023	March 12	March 18 – March 25	March 28
	2024	February 20	February 25 – March 4	March 8
Thuja plicata	2023	March 12	March 18 – March 25	March 28
	2024	February 20	February 25 – March 25	March 8
Juniperus sabina	2023	April 1	April 8 – April 17	April 20
	2024	March 5	March 11 – March 22	March 25
Juniperus communis	2023	April 26	April 30 – May 8	May 11
	2024	March 24	March 25 – April 14	April 20
Juniperus chinensis	2023	N/A	N/A	N/A
	2024	February 25	March 1 – March 11	March 15
Juniperys virginiana	2023	N/A	N/A	N/A
	2024	March 1	March 9 – March 17	March 20
Juniperus x pfitzeriana	2023	N/A	N/A	N/A
	2024	March 1	March 9 – March 17	March 20
Callitropsis nootkatensis	2023	N/A	N/A	N/A
	2024	February 25	March 1 – March 11	March 15
Chamaecyparis lawsoniana	2023	April 14	April 18 – April 25	April 28
	2024	March 16	March 18 – March 24	March 31

```
Score: 94.9367


Cupressus_arizonica DNPIDSCWRGDSNWDQNRMKLADCVVGFGSSTMGGKGGEIYTVTSSEDNPVNPTPGTLRY
Taxus_baccata            SCWRGDSNWGQNRMKLADCAVGFGSSTMGGKGGDFYTVTSAEDNPVNPTPGTLRY
                         *********.*********.*************::*****:**************

Cupressus_arizonica GATREKALWIIFSQNMNIKLQMPLYVAGYKTIDGRGAVVHLGNGGPCLFMRKASHVILHG
Taxus_baccata       GATREKALWXIFSQNXNIKLKMPLYVAGHKTIDGRGADVHLGNGGPCLFMRKVSHVILHG
                    ********* ***** ****:*******:******** **************.*******

Cupressus_arizonica LHIHGCNTSVLGDVLVSESIGVEPVHAQDGDAITMRNVTNAWIDHNSLSDCSDGLIDVTL
Taxus_baccata       LHIHGCNTSVLGDVLVSESIXVEPVHAQDGDAITMRNVTNAWIDHNSLSDCSDGLIDVTL
                    ******************** ***************************************

Cupressus_arizonica GSTGITISNNHFFNHHKVMLLGHDDTYDDDKSMKVTVAFNQFGPNAGQRMPRARYGLVHV
Taxus_baccata       GSTGITISNNHFFNHHKVMLLGHDDTYDDDKSMKVTVAFNQFGPNAGQRMPRARYGLVHV
                    ************************************************************

Cupressus_arizonica ANNNYDQWNIYAIGGSSNPTILSEGNSFTAPNESYKKEVTKRIGCETTSACANWVWRSTR
Taxus_baccata       AN
                    **
```

Fig. S1. A comparison of the amino acid sequences of the Cup a 1 protein from *Cupressus arizonica* and its homolog in *Taxus baccata*, performed using Clustal W, revealed 94.94% sequence similarity. Identical amino acids are indicated by an asterisk (*), conservative substitutions with similar chemical properties by a colon (:), semi-conservative substitutions with less similar but related properties by a dot (.), and the absence of similarity by a blank space.
